# Enabling the Industrial Internet of Things to Cloud Continuum in a Real City Environment

**DOI:** 10.3390/s21227707

**Published:** 2021-11-19

**Authors:** Fábio Henrique Cabrini, Filippo Valiante Filho, Pedro Rito, Albérico Barros Filho, Susana Sargento, Augusto Venâncio Neto, Sergio Takeo Kofuji

**Affiliations:** 1 Department of Electronic Systems Engineering, Polytechnic School, Universidade de São Paulo, São Paulo 05508-010, Brazil; filippo@usp.br (F.V.F.); alberico.castro@usp.br (A.B.F.); kofuji@usp.br (S.T.K.); 2 Instituto de Telecomunicações, University of Aveiro, 3810-193 Aveiro, Portugal; pedrorito@av.it.pt (P.R.); susana@ua.pt (S.S.); 3 Informatics and Applied Mathematics Department (DIMAp), Federal University of Rio Grande do Norte (UFRN), Natal 59078-970, Brazil; augusto@dimap.ufrn.br

**Keywords:** Industrial Internet of Things, IIoT platform, IIoT-to-cloud continuum, Helix Multi-layered, FIWARE

## Abstract

The Industrial Internet of Things (IIoT) is one of the most demanding IoT applications. The insertion of industries in the context of smart cities and other smart environments, allied with new communication technologies such as 5G, brings a new horizon of possibilities and new requirements. These requirements include low latency, the support of a massive quantity of devices and data, and the need to support horizontal communications between devices at the edge level. To make this feasible, it is necessary to establish an IIoT-to-cloud continuum distributing federated brokers across the infrastructure and providing scalability and interoperability. To attend this type of application, we present the Helix Multi-layered IoT platform and its operating modes. We report and discuss its real-world deployment in the Aveiro Tech City Living Lab in Aveiro, Portugal with functional and performance tests. We tested device-to-device communication across edge and core layers and also interconnected the infrastructure with one in São Paulo, Brazil, replicating the use of a global industry. The successful deployment validates the use of a Helix Multi-layered IoT platform as a suitable backend platform for IIoT applications capable of establishing the IIoT-to-cloud continuum. It also helps for the deployment of other applications in such a domain.

## 1. Introduction

Technological advances in diverse areas of knowledge enable the emergence of complex, responsive, resourceful, and capable smart environments that can supply better conditions for human activities. The Internet of Things (IoT) stands out as one of the enabling technologies responsible for this transformation and for the emergence of applications such as smart meters, logistics systems, and fleet monitoring, which assume millions of devices connected in the same coverage area. The classification of IoT applications in categories follows strict requirements, for instance, the demand for ultra-low latency (between 1 and 10 ms) by the Internet of Medical Things (IoMT) [1], intelligent transportation system (ITS) [2], and the Industrial Internet of Things (IIoT) [3], which allow the categorization under this label. Furthermore, the overall infrastructure must fully meet the application requirements to accommodate the high density of devices, low latency, and the communication between devices at all infrastructure layers while promoting interoperability, highlighting the need to rethink conventional cloud computing for efficient support for IoT applications.

The IIoT architecture [3] highlights significant technological challenges at application, communication, and physical layers. Applications (e.g., smart factory, smart plant, and smart supply chain) present important factory and process automation scenario requirements that follow a production cycle involving stages, such as receiving raw materials, preparing machines, producing parts, counting, quality analysis, packaging, distribution logistics, and personnel management. Many processes involve sensors, actuators, and augmented reality systems interconnected to a communication and computing infrastructure that acts in the control and supervision of the processes. Low latency in operations and high availability of services, together with a secure and resilient technological infrastructure, can add tremendous value to the entire production chain.

Each of the proposed layers presents excellent improvements, such as machine learning to achieve high assertive levels while processing information and the addition of modern communication technologies based on fifth generation (5G) cellular networks with virtualized infrastructure and edge computing [3]. However, it is necessary to advance towards the use of IoT platforms that are capable of offering excellent adherence to new communication and processing technologies, integrating a large number of applications in multiple domains, or distributed industrial plants, while integrating the urban infrastructure and offering mechanisms that enable horizontal and vertical communication, integrating cloud, fog, and edge layers present in the production ecosystem and cities.

The interaction between edge and cloud systems, denoted as the IoT-to-cloud continuum [4], enables a distributed computing approach to fulfill the heterogeneous demands of different IoT verticals. The proximity between edge datacenters to IoT devices is vital to significantly reduce the latency of decision-making applications, drastically save bandwidth by avoiding data transfers and communication overhead from IoT devices to central cloud datacenters, and to protect privacy, among other benefits [5].

This study aros eby researching the benefits of the IoT-to-cloud continuum over industrial systems, which we baptized the IIoT-to-cloud continuum. Those IIoT systems can harness innovative technologies to empower industrial applications while enabling high system availability, on-site computing performance, enhanced network efficiency, and security/privacy at the same time. The main contribution behind our research lies in the design of the Helix Multi-layered platform, which offers an IIoT service platform and settles a federation of context brokers across cloud, fog, and edge layers (i.e., the IIoT-to-cloud continuum).

According to the NECOS [6] common management and orchestration (MANO) ecosystem, the Helix Multi-layered platform is automatically containerized within the IIoT-to-cloud continuum datacenter servers. Thus, the Helix Multi-layered platform goes beyond the state of the art by extending the horizontal digital continuum (mostly presented at central cloud approaches, e.g., the FIWARE platform) towards the fog and edge layers through a horizontal and vertical federation of context brokers. Hence, the Helix Multi-layered platform establishes an IIoT-to-cloud continuum towards managing and processing advanced context information from multiple tenants and reducing traffic and latency rates in communication between consumers and context generators through open standards with ensured interoperability and adherence to the upcoming 5G ecosystem approach.

In order to achieve a highly accurate validation approach of our proposal, we prototyped the Helix Multi-layered platform in a real-world testbed at the Aveiro Tech City Living Lab ecosystem (https://aveiro-open-lab.pt/citymanager, accessed on 23 August 2021) [7,8]. The outcomes suggest that the Helix Multi-layered platform enables a reduction in latency and information traffic costs between context producers and context consumers located in industrial plants, along with context consumers distributed and connected to the cities’ 5G network infrastructures. The Helix Multi-layered platform operation shows results for machine-to-machine (M2M) and people-to-machine (P2M) interaction intermediated by a federation of context brokers, which are instantiated at fog- and edge-layer datacenters.

The organization of this article is as follows. Section 2 elucidates the most significant endeavors concerning solutions tailored to the IIoT-to-cloud continuum. Section 3 presents the Helix Multi-layered platform in detail, highlighting available operating modes tailored to brokers’ interconnection. Section 4 presents the real-world testbed implementation at the Aveiro Living Lab, along with a set of tests we conducted. Section 5 describes the performance tests in the laboratory and real-world environments. We discuss the results in Section 6. Finally, Section 7 proceeds with the conclusions and a list of future work.

## 2. Related Works

The research presented in this article aimed to show an open and interoperable platform to establish the IIoT-to-cloud continuum. Our state-of-the-art study of available technologies that aim to create a continuum between IoT, edge, fog, and cloud layers revealed efforts in several directions.

FIWARE (https://www.fiware.org, accessed on 8 September 2021) is a complete and modular open-source platform, maintained by the FIWARE Foundation, for smart environments such as smart cities, smart manufacturing, and smart robotics. The FIWARE modules are known as generic enablers (GEs) and use next-generation service interfaces (NGSI) and next-generation service interfaces linked-data (NGSI-LD) information models. The authors of [9] present the FIWARE-based architecture for industrial data space (IDS) aimed at a cloud-based infrastructure. They have shown a real testbed in an industry 4.0 scenario to analyze data from a milling machine and a coordinate-measuring machine (CMM) system to perform predictive machine maintenance and to detect defects in production processes. The authors present the security architecture by enforcing security aspects. Despite this, the implementation was cloud-centric and served only one industrial plant. There was no mention of a cloud continuum for things, fog computing, or any other feature to allow for distributed operation, reduced data traffic, or even latency.

Within the FIWARE ecosystem, new initiatives appeared to manage context information to the regions of fog and edge, including initiatives such as Cepheus, a discontinued generic enabler that acted as a broker and performed complex event processing (CEP) in edge environments, in addition to the IoT broker and IoT discovery GEs. The IoT broker and IoT discovery were substantially modified to give rise to FogFlow, which was incorporated into the GEs catalog of the FIWARE ecosystem [10,11].

FogFlow, a framework-like technology developed by NEC, works as a module to FIWARE-based platforms offering orchestration capabilities for processing modules and integrating geo-distributed IoT devices connected via edge to the cloud. It supports major IoT protocols in an approach that seeks to reduce latency [10,11] and provides an NGSI-based programming model using data flow and declarative suggestions to allow easy programming, interoperability, and scalability. It also presents an architecture of three main layers: service management, context management, and data processing. Workers and IoT brokers are essential elements to the operation on regions of fog and edge. According to the developers, IoT brokers manage some of the context information and record it in the IoT discovery [12]. IoT discovery manages all recorded context availability information, including ID, entity type, list of attributes, and metadata. It allows other components of the platform to consult and sign their context availability information via NGSI messages.

The work from Cheng [10] presents a comparative study between the IoT broker from FogFlow and the Orion context broker used on the FIWARE platform. It concludes that the IoT broker present in FogFlow presents a higher performance in terms of processing. FogFlow’s IoT broker keeps only the most recent value of each context entity and saves it directly in the system memory and not in a database as in the Orion context broker [12]. FogFlow emphasizes vertical communication from cloud to edge rather than horizontal communication; it does not specify a set of operating modes to support direct device-to-device communications through the edge or fog layers.

Munoz-Arcentales et al. show in [13] the implementation of an architecture for access and usage control in industry and smart cities using the European open-source platform FIWARE. This represents a real-life use case in the food industry, demonstrating the ability of FIWARE security components to provide a flexible and robust architecture to manage the usage control in data sharing ecosystems.

The authors of [14] deal with the integration of cloud-edge-IoT, but focusing on orchestration and configuration of application services and data flows for industrial cyber–physical systems. Its basis is the Arrowhead framework. The platform uses a service-oriented architecture (SOA) and allows for services’ interoperability. They report an IoT platform integration but do not mention which platforms or protocols are in use. The focus is on composing application services, orchestrating them along the continuum. They also report two industrial use cases for evaluation.

Recent works, such as [15,16], address the topics of cloud-to-things or the cloud-edge continuum, offering detailed surveys about the concepts and their advantages. Both works report dozens of simulators for IoT applications and resource management along this continuum. Several of the works mentioned are complete frameworks and deal with complex scenarios involving interoperability with other systems and detailed performance and resource consumption analysis. However, they lack real-world implementations and evaluation.

Xu et al. [3] present a detailed study of control, network, and computing issues. They suggest two sets of three-dimensional analyses to adopt computing and networking technologies. In the case of networks, they specify the use of 5G, machine-to-machine communication, software-defined networks (SDN), cloud-computing technologies, hybrid clouds, and computing platforms to address problems in IIoT applications.

Lavassani et al. [17] present the use of machine learning at the fog level to perform raw data processing from wireless sensor networks (WSN). Data are sent via message queuing telemetry transport (MQTT) to the ThingsBoard IoT platform located in the cloud, showing that the approach significantly reduces the amount of data sent to the cloud. This approach applies machine learning in fog to minimize the flow of information generated in the WSN to the cloud. The document does not suggest the use of standard data models at fog and edge levels. It also does not demonstrate the exchange of information between devices present on different edges and experiments performed on real hardware.

Benomar et al. [18] implement a fog-based monitoring system for industrial equipment monitoring using a real-world scenario. The platform also integrates industrial wireless sensor networks (IWSN) through its IoTronic interface, which is responsible for connecting the Stack4Thing platform to access gateways through the control channel, service forwarding, and virtual networking. Although the approach reduces the flow of data sent to the cloud, we found no evidence of direct communication between devices located on different edges or mediated by fog access gateways. The use of standardized data models at fog or edge levels was also not observed.

Vítor et al. [8] present the architecture overview of the Aveiro Tech City Living Lab current data platform and the flow of the data on the gathering and the export to the third-party users. In particular, it analyzes the data flow of the collected data and the data retrieval, considering the IoT agents, the real-time data transport, the access and authentication, and the data processing and persistence. The results concerning the amount of data gathered and examples of data show how that platform serves as a base for developing new applications and introduces an intelligent layer for future predictions and actuations.

Cabrini et al. [19] introduced a new IoT platform, denoted the Helix Multi-layered platform, which differentiates itself from other solutions to deploy the IoT-to-cloud continuum due to the process and distributed management of complex and straightforward context information across the edge, fog, and cloud layers. When running in edge datacenter premises, the Helix Multi-layered platform performs data processing as close as possible to the IoT nodes in charge of generating and/or consuming context information, thus enabling reduced rates of latency and information traffic on the network. Helix also promotes interoperability with other FIWARE platforms and is ready for the Connecting Europe Facility (CEF) (https://ec.europa.eu/inea/en/connecting-europe-facility, accessed on 9 September 2021).

To address the lack of the IIoT-to-cloud continuum in previous approaches, we present the Helix Multi-layered approach. Moreover, it offers complex context information processing at the edge layer while maintaining a lightweight approach, which is a novelty compared to the work in the literature. Finally, to the best of our knowledge, previous NGSI-based approaches do not feature device-to-device communication into a continuum composed of context brokers to enable edge-to-edge, edge-to-cloud, fog-to-cloud, and cloud-to-cloud communication applied into real-world infrastructure in a smart city for IIoT applications.

## 3. Helix Multi-Layered Platform Description

The Helix Multi-layered platform is a general-purpose IoT backend platform that acts as a distributed framework at the cloud, fog, and edge layers, establishing an interoperable IoT-to-cloud continuum based on open standards.

The platform implements an advanced multi-tenancy mechanism that ensures the operation of multiple IoT applications, providing the necessary isolation for applications at all layers of the network. In addition, the platform has a module, called a slice descriptor, that integrates with MANO platforms, responsible for instantiating, orchestrating, and managing its modules in the SDN infrastructure.

Helix establishes a continuum capable of serving multiple IoT applications across its entire structure, optimizing the use of distributed context brokers through the horizontal and vertical federation mechanism at the infrastructure layers. This approach allows the use of advanced features such as conditional subscriptions, relationships, notifications, geoprocessing, and the location of context providers, using the well-known public/subscriber method. Furthermore, it enables the integration and use of multiple protocols of IoT, necessary for IoT applications with different requirements and purposes.

Helix implements a container-based approach that enables more efficient use of the computing resources available in the infrastructure by deploying cloud context information management (CCIM) based on the swarm of Orion context brokers into the Helix Nebula module. The platform also uses Orion-based Helix brokers in the fog and edge layers, running in single or clustered mode to meet the requirements of IIoT applications. It presents the slice descriptor module that simplifies integration with SDN on MANO platforms, such as network slices that promote connection, resource reservation, and respective application isolation.

Helix also specifies various operational modes applied to bidirectional communication between producers and consumers of context information in distributed IIoT scenarios across cloud, fog, and edge layers. It offers compatibility with NGSIv2 and NGSI-LD protocols, making it simple to manage context information at all levels.

### Helix Operating Modes in the Context of IIoT Applications

In this section, we highlight the main modes of operation adhering to IIoT scenarios integrated into smart cities, such as remote control, remote sensing, logistics, fleet management, automated industrial plant, augmented reality, and monitoring parameters such as overall equipment effectiveness (OEE) and key performance indicators (KPIs) used by decision-makers.

**Figure 1:**
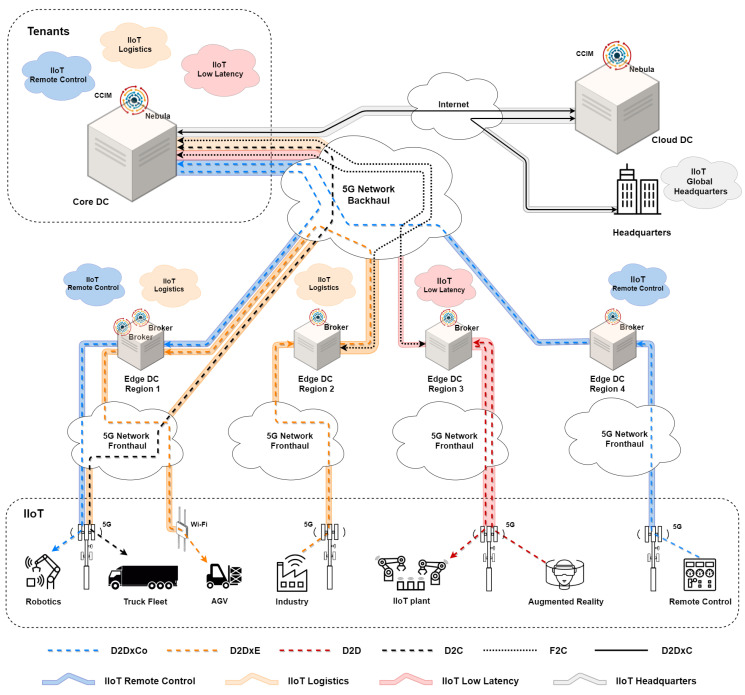
Helix Multi-layered operating modes for IIoT.

Figure 1 presents an architecture that distributes three IIoT applications managed by the Helix platform in a distributed scenario in a pseudo-city served by a 5G network. The infrastructure has three types of datacenters (DC) at the cloud, core, and edge layers that use a virtualized infrastructure manager (VIM) and MANO platforms, interconnected by an SDN infrastructure.

The Helix platform forwards the requirements of IIoT applications to the MANO platform responsible for instantiating the computational resources and establishing the cloud slices for isolating the applications.

First, we introduce the remote management of robots in industrial plants through the device-to-device cross core (D2DxCo) operating mode. Figure 1 shows a robot associated with the Helix broker located in Region 1 controlled by a remote control panel connected to the Helix broker in Region 4. The CCIM located at the core datacenter interconnects the remote Helix brokers. This mode makes it easy to connect IoT devices in different regions served by the same telecommunication company.

Second, we present two logistic applications. The first app monitors the truck fleet through a cloud-centric approach. Trucks send sensor and geolocation information directly to CCIM using 5G base stations and the device-to-cloud (D2C) operating mode.

The information transmitted to the datacenter by the SDN network is stored and processed to find better routes with the aid of geoprocessing, historical data analysis, and zoning rules, favoring an efficient and safe operation, in addition to optimizing the transport of inputs and products and reducing the negative impact of this activity on the urban perimeter.

The second logistics app highlights automated guided vehicles (AGV) in industrial plants and logistics centers, with artificial intelligence algorithms integrated into the Helix platform control AGVs. Device-to-device cross edge (D2DxE) mode provides low-latency bidirectional communication between AGVs located in industrial plants and distributed logistics centers across the city.

Figure 1 shows an AGV connected via Wi-Fi technology and associated with the Helix broker in Region 1. The AGV exchanges information with the control center located in Region 2. This example also demonstrates the use of the fog-to-cloud (F2C) operating mode responsible for merging data through advanced relationship techniques and conditional subscriptions that enable the reduction of the volume of parameters notified to the CCIM.

Third, an IIoT application with ultra-low latency requirements is presented, evidenced by the integration of the automated production belt into an augmented reality system. The Helix broker connects the systems by being located close to the industrial plant. The device-to-device (D2D) operating mode establishes communication between the systems and can reach latencies below 10 ms.

Finally, we present the interconnection of the industrial plant with the global headquarters through the device-to-device cross cloud (D2DxC) operating mode.

## 4. Towards a Helix-Empowered Real-World Testbed Prototyping

Smart cities act in synergy with IIoT systems, contributing positively to the consolidation of the industrial production ecosystem. The displacement of employees within the urban perimeter, the transportation of products and raw materials, the use of manufacturing execution systems (MES), and the interconnection of manufacturing units distributed in the city corroborate the success of the IIoT.

### Description of the Scenario

The design of the Aveiro Tech City Living Lab (https://www.aveirotechcity.pt, accessed on 25 August 2021) followed three main objectives: (i) to build a communication infrastructure connected by fiber optics, integrating a multi-protocol communication network with multi-radio terminals; (ii) to implement a sensing platform capable of understanding citizens’ behavior within the city and providing new solutions towards efficient traffic management, intelligent transportation system, and citizens’ safety; and (iii) to provide an open platform for third-party partners to test their protocols, mechanisms, prototypes, or explore the collected data.

**Figure 2:**
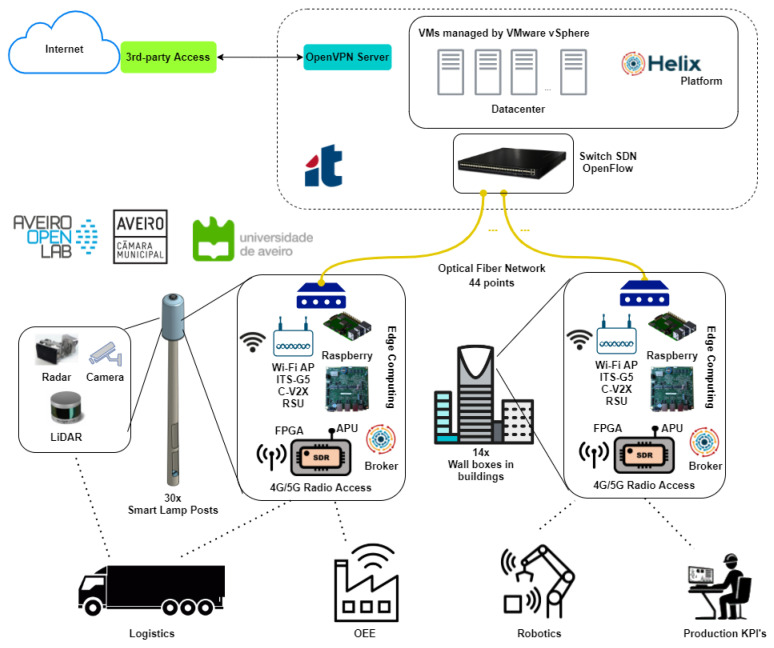
Aveiro Tech City Living Lab infrastructure.

The description of the structure of the living lab presents itself in the following manner (Figure 2): (i) access and edge platform, supported on fiber technology, edge computing, high data-rate SDN network devices, and radio units mounted in smart lampposts and buildings; (ii) sensing platform, with static and mobile sensors in vehicles collecting various forms of data, such as mobility and environment data; (iii) backhaul and core platform, consisting of the datacenter with core network devices and servers; and (iv) backend and data platform, composed by the core data platform with the broker, database, and data processing. The backend and data platform were focus of this study.

The Helix Multi-layered platform was deployed and evaluated in this city platform to identify its potential to support services and applications that require operation in an edge environment.

The first step was to set and exchange experiences between the teams from the University of São Paulo, the Federal University of Rio Grande do Norte, and the Instituto de Telecomunicações (University of Aveiro). The second stage began from that point on, which defined a test scenario to validate the installation process, configuration, and analysis of the operating modes. Then, we performed the platform behavior analysis on the experimental infrastructure available at the laboratory of the group of Network Architectures and Protocols of the Instituto de Telecomunicações. Finally, the selection, specification, and execution of the proof of concept (PoC) meeting the services present in the city of Aveiro was done.

The second step comprised the evaluation tests. It started with the instantiation of a virtual machine with Helix Nebula components in the datacenter of the Instituto de Telecomunicações. The virtual machine had the following configurations: Intel Xeon, four virtual processors (vCPUs), 3 GHz, 2 GB RAM, 100 GB HDD, Linux Ubuntu Server 18.04 LTS operating system, and a docker engine.

We used two PC engine accelerated processing unit (APU) system board servers with x86 cores to instantiate the Helix brokers that took on the role of edge servers. Edge servers featured the following configurations: AMD APU2 Embedded G Series GX-412TC 1 GHz quad Jaguar Core 64 bit, 4 GB RAM, 28 GB HDD, and a Voyage Linux operating system.

We interconnected the servers via fiber-optic links and switches operating at 1 Gbps. We also used Wi-Fi and gigabit Ethernet links to connect the Raspberry Pi 4B with the APUs to set the stage for end-to-end experiments.

The Raspberry Pi 4B ran the Tx and Rx software modules emulating the IoT devices. For the correct synchronism of IoT modules and other computational components, it was necessary to use the network time protocol (NTP) service.

The experimental scenario made it possible to validate the D2D, D2DxE, F2C, D2C, and D2DxCo operating modes available on the Helix Multi-layered platform. The tests also made it possible to assess the vertical and horizontal federation of Helix brokers, analyzing the latency and operation of the current resources available on the Helix platform.

The third step used the infrastructure described in Figure 2, which presents the high-level architecture of the Aveiro Tech City Living Lab. The architecture consists of the following components: datacenter, fiber optic links, internal pole components, and the Helix backend platform modules, described at [7].

**Figure 3:**
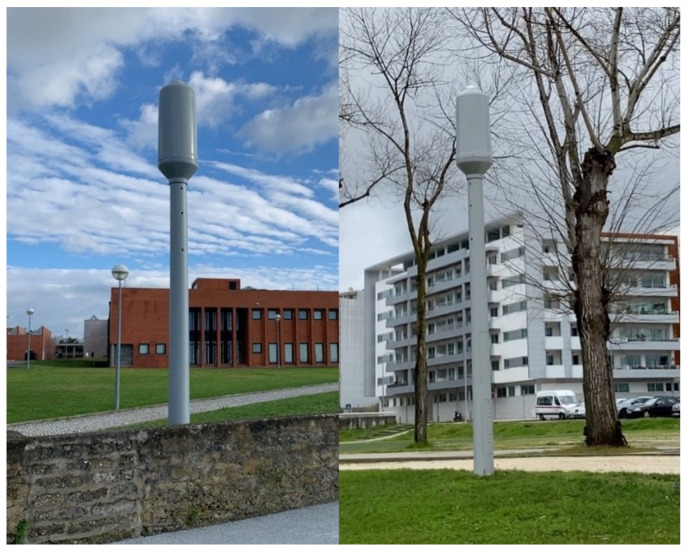
Infrastructure at Aveiro.

Figure 3 shows stations P1 and P22 in perspective, showing their location on the University of Aveiro campus and in the Congress Center. The poles have in their structure an upper dome that houses the APU and Raspberry Pi responsible for the execution of Helix brokers and IoT modules Tx and Rx, in addition to other computational elements, sensors, and actuators.

**Figure 4:**
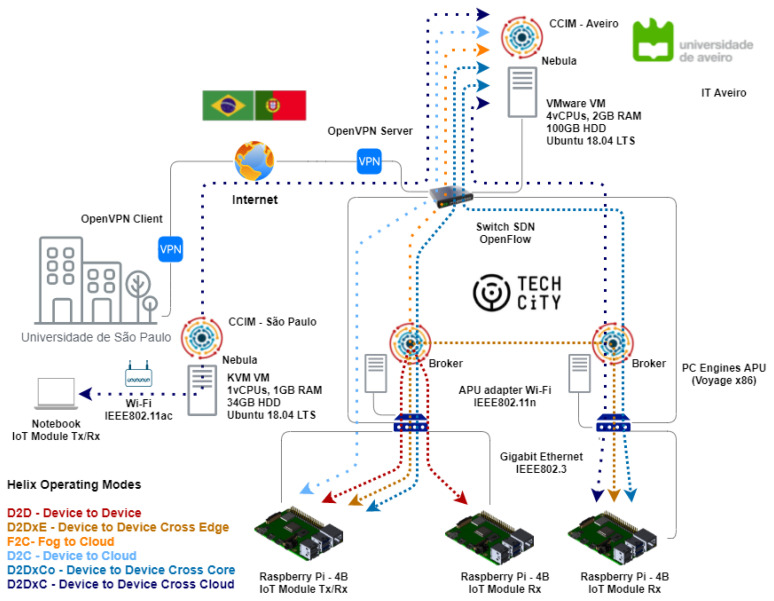
Infrastructure used to evaluate the Helix Multi-layered platform at the Aveiro tech city living lab.

Finally, we established a virtual private network (VPN) to connect the infrastructure of Aveiro Tech City Living Lab to the Helix Nebula module at the University of São Paulo, which allowed us to evaluate the D2DxC operating mode, as shown in Figure 4.

We carried out a complementary experiment to validate the capacity of the Helix Multi-layered platform to transmit large volumes of data in the NGSIv2 standard through a transcontinental connection. We analyzed the D2DxC operating mode, where IoT frequency probes located in Aveiro sent information collected on smart poles P1 and P22 to a Helix instance in São Paulo, Brazil.

**Figure 5:**
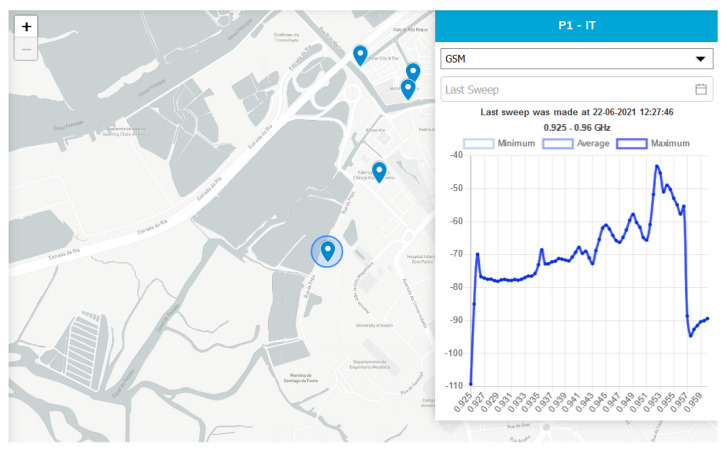
GSM signal frequencies from the spectral probe at P1.

The IoT frequency probes operate with the following frequency bands: global system mobile (GSM), digital cellular system (DCS), universal mobile telecommunication system (UMTS), multichannel multipoint distribution service (MMDS), digital television (DTV-590), and DTV-750. Figure 5 presents one of the Aveiro Tech City Living Lab’s dashboard tabs, showing the power levels of the GSM signal frequencies in the 925 MHz to 960 MHz frequency range in the vicinity of pole P1.

The Helix brokers, instantiated in the APUs of pole P1 and P22, received notifications generated by the IoT probes. We also made changes to the IoT device source code to support NGSIv2 messages.

## 5. Performance Analysis

**Figure 6:**
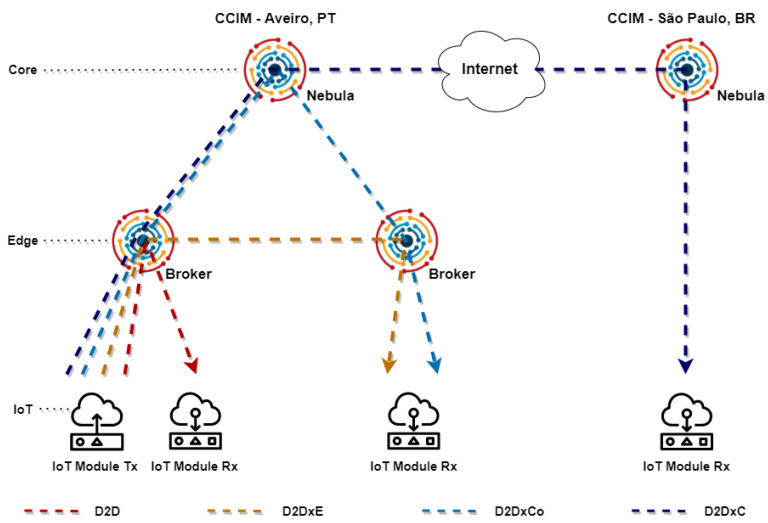
Topology for evaluating D2D, D2DxE, D2DxCo, and D2DxC operating modes.

After realizing the functional tests using real-world data gathered from the spectral probes, we conducted a set of tests using software modules to evaluate the performance of our solution. We tested D2D, D2DxE, D2DxCo, and D2DxC operating modes. Then, we tested the communication between Aveiro, Portugal and São Paulo, Brazil. Figure 6 highlights the logical topology of the experimental scenario and operating modes evaluated. We used the Raspberry Pi inside the smart poles in the infrastructure of the city of Aveiro for the IoT devices, as depicted in the previous item. In Brazil, we used a standard x86 notebook to run the software IoT module, configured with an Intel Core i7 6500U microprocessor, 8 GB DRAM, and running Ubuntu Linux 20.04 LTS.

We measured the one-way delay (OWD) for each operating mode. The software IoT module sends a batch of 1000 NGSI update messages per test. We repeated each test at least three times in different moments to check if the values were consistent. We also varied the interval between update messages as 100 ms, 10 ms, and 1 ms. The last one corresponds to a more stressing condition like that preconized for 5G environments.

Remarkably, we performed the tests in a real-world condition, i.e., our tests ran in parallel with other applications in the city infrastructure, e.g., the spectral probes mentioned before. We did not shut down applications or use any bandwidth or resource reservation. Thus, we present the obtained results and not just expected ones, carrying out a more profound analysis and discussion of such implementation.

Previously, we tested D2D, D2DxE, and D2DxCo operating modes in a laboratory environment inside the Aveiro Tech City Living Lab infrastructure, using the same configurations as the smart poles, but this time with fully dedicated resources. Thus, we can compare an ideal scenario with the real one.

**Figure 7:**
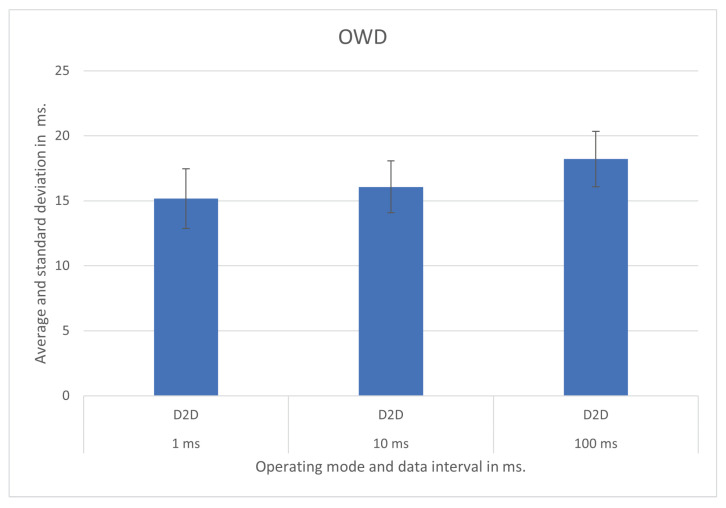
D2D operating mode test results.

**Figure 8:**
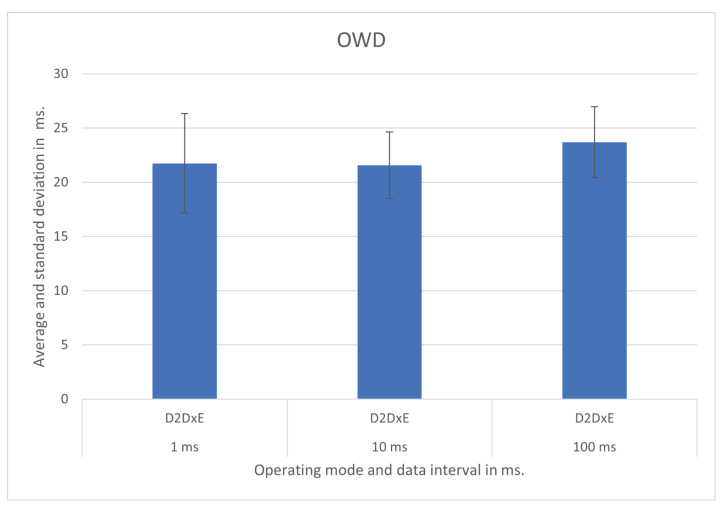
D2DxE operating mode test results.

**Figure 9:**
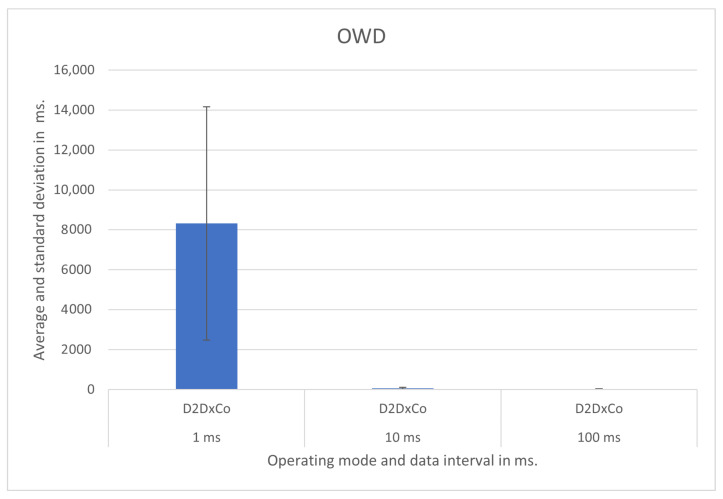
D2DxCo operating mode test results.

**Figure 10:**
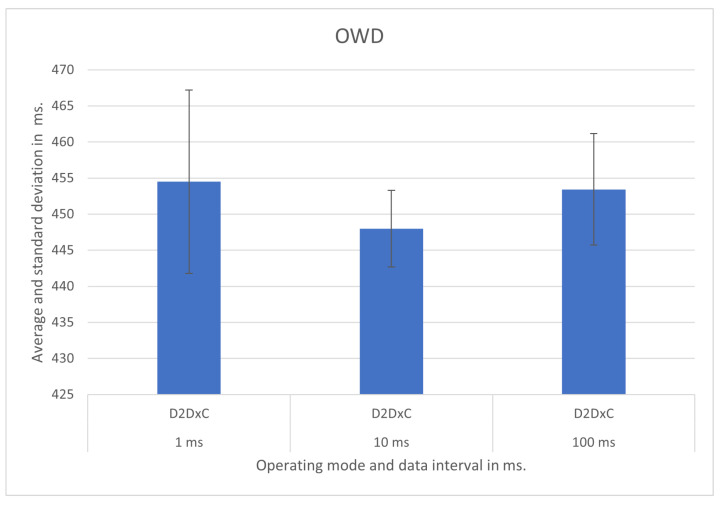
D2DxC operating mode test results.

**Figure 11:**
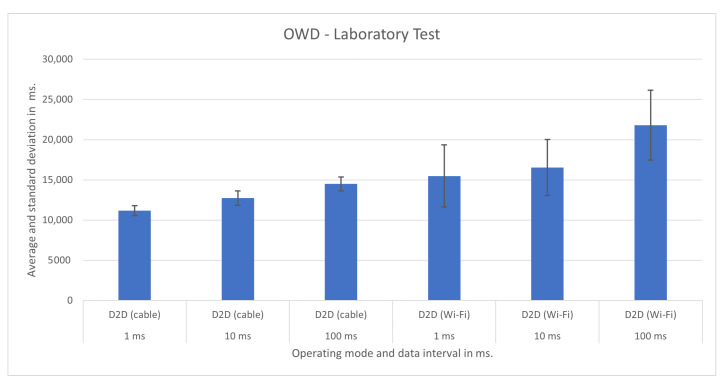
D2D operating mode test results in laboratory.

**Figure 12:**
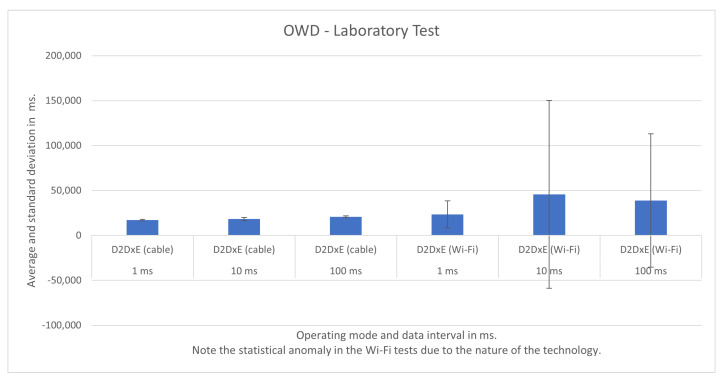
D2DxE operating mode test results in laboratory.

We show the test results for each operating mode and respective scenario in Figure 7, Figure 8, Figure 9, Figure 10, Figure 11 and Figure 12, highlighting the mean OWD and standard deviations. Naturally, considering individual messages, we obtained OWD times below and above the standard deviation, but there was no message loss during the tests, so we treated them as mere outliers.

### 5.1. Device to Device—D2D

In the D2D operating mode, tested using different IoT modules running on smart pole P1, we obtained OWDs of about 15 ms, 16 ms, and 18 ms for 1 ms, 10 ms, and 100 ms update messages interval, respectively. The graphical of the results with the respective standard deviation is in Figure 7.

In this test, the connection between the Raspberry Pi that runs the IoT modules and the APU that runs the Helix broker was through cabled gigabit Ethernet.

### 5.2. Device-to-Device Cross Edge—D2DxE

In the D2DxE operating mode, evaluated between smart poles P1 and P22, we obtained OWDs of about 21 ms, 21 ms, and 23 ms for 1 ms, 10 ms, and 100 ms update messages’ intervals, respectively. The graphical of the results with the respective standard deviation is in Figure 8.

In this test, the connection between the Raspberry Pi that runs the IoT modules and the APUs that run the Helix brokers was through cabled gigabit Ethernet. The link between the brokers was optical fiber gigabit Ethernet passing through a layer two switch.

### 5.3. Device-to-Device Cross Core—D2DxCo

We also evaluated the D2DxCo operating mode between smart poles P1 and P22. Nevertheless, this time, the broker in the core datacenter mediated the connection between edge layer brokers. For intervals of 10 ms and 100 ms between updates, the OWD was 63 ms and 30 ms, respectively. However, in this scenario, the stressing update interval of 1 ms overloaded the IoT devices and the reception broker, resulting in a mean OWD of 8 s. The graphical presentation of the results with the respective standard deviation is in Figure 9.

In this test, the connection between the Raspberry Pi that runs the IoT modules and the APUs that runs the Helix brokers was through cabled gigabit Ethernet. The links among all brokers at the core and edge layers were optical fiber gigabit Ethernet.

### 5.4. Device-to-Device Cross Cloud (Aveiro, PT—São Paulo, BR)—D2DxC

We tested D2DxC through a communication between P22 at Aveiro’s infrastructure with a notebook running the IoT module in São Paulo, Brazil. The infrastructure in Aveiro remained the same, while the edge broker in São Paulo ran in the USP datacenter. The link between the IoT module and the USP datacenter was through the public Internet. This was the same for the one between USP and Aveiro’s datacenters but using a VPN in this case. We obtained 450 ms for OWD in the three update intervals in this scenario. The graphical presentation of the results with the respective standard deviation is in Figure 10.

### 5.5. Laboratory Testbed

We proceeded with a laboratory testbench using the same equipment present in the smart poles connected through a cabled gigabit Ethernet network. In these tests, we counted with exclusivity for the equipment with no other application running in parallel. In this controlled environment, we made several functional and performance tests. We ran batteries of 100 update messages and 1000 update messages and validated the tests we made in the city infrastructure.

Besides standard non-persistent hypertext transfer protocol (HTTP) connections, we also implemented persistent connections in the first hop, i.e., the communication between the IoT device and its edge broker. Connections for the notifications from this broker to the others were non-persistent. We noticed a performance gain in this situation, but initial results trying persistent connections along the pathway met several bottlenecks, so we did not replicate these tests in the production environment.

In the following, we present the results for the D2D, D2DxE, and D2DxCo operating modes in Figure 11 and Figure 12. When using the cabled connection, the D2D and D2DxE operating modes had slightly better results than the ones obtained in the production scenario. It was as expected, as there was no concurrency of other services in the computers running the brokers.

In the laboratory, we also tested the Wi-Fi connection (IEEE 802.11n), and the results were much worse, even worse when compared to the ones obtained in the city infrastructure. It is not possible to overlook the impact of communication technology on performance. Due to the nature of Wi-Fi, some updates were so delayed that we obtained a standard deviation higher than the mean OWD itself, as is shown in Figure 12.

## 6. Discussion

Both functional and performance analyses showed that the Helix Multi-layered platform is a feasible solution for IIoT, smart cities, and similar applications. We successfully routed context information through the near edge and the city’s cloud datacenter. Moreover, we integrated and tested across the ocean to other countries, cloud datacenters, and internet service providers, replicating the original data. It corresponds to a scenario where a company’s global headquarters can monitor regional branches. In this last case, connections between core datacenters used a VPN.

Table 1 shows a synthesis of the obtained results in the city’s infrastructure.

The results show that, although the platform allows the establishment of a continuum, the choice of equipment and media must be judicious. For instance, Wi-Fi IEEE 802.11 n presented significant delays and was very unstable in the laboratory tests. If an application needs Wi-Fi, it must consider more modern versions of the protocol with larger bandwidth and quality-of-service (QoS) mechanisms, such as Wi-Fi6 (IEEE 802.11 ax). Edge equipment presented processing and memory bottlenecks when the update interval was as small as 1 ms in more complex operating modes (e.g., D2DxCo), implying more hops to replicate update messages across the brokers due to the presence of multiple opened simultaneous TCP connections. The platform did not lose any update message. We tested “worst-case” scenarios not implementing conditional subscriptions, which may drastically reduce the number of messages sent.

### 6.1. Comparison with Aveiro’s Present Architecture Using MQTT

The current implementation at the Aveiro Tech City Living Lab used an independent MQTT broker at each IoT and edge device. It used an MQTT bridge in the cloud to aggregate all information. In this way, it was possible to consume data of every edge broker using subscriptions processed in the cloud. All the computational analysis happened at the cloud layer. Moreover, edge services can subscribe to other edge devices directly using the link to the local brokers, enabling services to run on the edge. However, the NGSIv2 protocol is not available at the edge of the infrastructure, even though the information uses JSON format.

Using the Helix Multi-layered platform, it was possible to work in the same way. However, we obtained an advantage by using conditional subscriptions because of the edge broker and passing through all the layers in the continuum. The conditional subscriptions set triggers to forward the data. It can deal with more complex analysis, such as geolocalization, for instance. It enables the reduction of data volume transferred to the cloud considerably.

To allow interoperability with other systems and platforms, the Aveiro Tech City Living Lab connected the MQTT bridge with FIWARE’s Orion context broker in the cloud, making the city’s data available as properly configured [8]. The use of the Helix platform in the present work allowed for a more scalable and distributed implementation while maintaining compatibility with the current scenario.

The lightweight approach of the Helix broker allows the implementation of the open standard of NGSIv2 protocol already from the IoT and edge layers, provided that the IoT device supports hypertext transfer protocol secure (HTTPS). Thus, there was no need for extra work to interoperate with other platforms at any architecture layer besides conditional subscriptions, even acting as a context provider for the city’s data and receiving data from other context providers, e.g., climate and public transport. If IoT devices need to use MQTT, the Helix platform can convert the data to the NGSI format at any architecture layer to take advantage of it. The Helix Multi-layered platform can also aggregate data at any layer, providing for more complex distributed systems. The use of NGSI protocols makes it easier to adopt smart data models (https://smartdatamodels.org, accessed on 4 October 2021). These free and open models enable portability among different IoT vertical applications, including smart manufacturing, smart robotics, and smart cities.

Another advantage brought by the Helix platform is to allow the bidirectional device-to-device communications through the local edge broker and a pool of edge brokers. It corresponds to D2D and D2DxE operating modes. This is possible since the Helix Multi-layered platform implements a vertical federation of context brokers and a horizontal federation. The Helix Multi-layered platform allows the device-to-device communications to flow through the necessary path across the context brokers’ federation. It also matches the concept of fog computing presented by the Industry IoT Consortium and IEEE in [20].

The Helix platform also allows context-broker clustering, dealing with abrupt demand alteration for context messages.

### 6.2. Applications in Other IoT verticals

The Helix Multi-layered architecture is also feasible for other IoT verticals like IoMT and ITS. An increase in the demand for IoMT is due to people’s longer life expectancy and the emergence of new technologies for e-health and m-health. Critical applications for life care and maintenance in emergencies are sensitive to latency, jitter, and service unavailability. In this context, the existence of medical network slices (MNS) gains importance for providing services beyond teleconferences or augmented reality glasses, which have enhanced mobile broadband (eMBB) requirements. It is necessary to meet a new class of applications with ultra-reliable low-latency communication (URLLC) and massive machine-type communications (mMTC) requirements. The use of MNS can improve sensitivity in robot-assisted remote surgical procedures and can provide better monitoring and assistance to procedures performed in m-health environments, such as activities performed by paramedics in connected ambulances, monitoring, and alerting on home care or wearables [1].

The Helix Multi-layered architecture can establish a continuum between the deepest layers of the edge and can enable the processing of context information from IoMT applications. In this way, the health system’s control and monitoring centers receive reports of the events quickly. This type of approach significantly reduces network overhead and speeds up decision-making or activating any asset present anywhere in the network. It is also possible to expand the brokers’ processing capacity through the scaling obtained by clustering the architecture modules at any network layer. This type of resource allows brokers to process many requisitions or meet temporary demand peaks arising, for example, from events caused by major accidents.

Intelligent transportation system is becoming more feasible as 5G networks are making it possible to integrate a large number of services, add value, and expand the capabilities of vehicles in the exchange of information with the vehicle-to-infrastructure (V2I) environment and vehicle-to-vehicle (V2V) and vehicle-to-pedestrian (V2P) communication. That is, they can exchange information with anything (V2X) as presented by [21].

The integration of ITS with 5G networks is essential to the modern world, such as connecting multiple vehicles that enable collaborative and more accurate decision-making corroborating action planning. This integration will help to solve problems related to the transmission rate of the current third- and fourth-generation networks; connection stability; reliability due to the high speed of the vehicles; and the collection of numerous information from sensors, such as radars and LiDARs, in addition to improving security, making these systems more convenient, secure, fast, and efficient, as presented by [2].

In this context, we presented the Helix Multi-layered platform as an alternative backend platform capable of supporting future applications for ITS, offering, together with the MANO platforms, a continuum that makes it possible to meet the needs of applications due to its architecture in multiple layers and their native integration into the infrastructure of current 5G networks.

## 7. Conclusions and Future Work

In this article, we proposed an integrated architecture and presented experiments with the Helix Multi-layered platform at the Aveiro Tech City Living Lab to validate the operating modes used to establish an IIoT-to-cloud continuum integrating a smart city’s infrastructure to the industry’s ecosystem.

The first set of experiments, aimed at reproducing the real-world scenario, took place in the network laboratory of the Instituto de Telecomunicações de Aveiro, promoting the total isolation of the platform without the presence of competing applications on the edge’s APUs and IoT devices. The experiments made it possible to instantiate the Helix Nebula module in the datacenter of the Telecommunications Institute of the University of Aveiro, using the setup of APUs with Helix brokers and Raspberry Pi, which played the role of IoT devices. The experiments evaluated the OWD time between edge Helix brokers and IoT devices.

We tested two types of fronthaul, gigabit Ethernet and Wi-Fi (IEEE802.11n), making it possible to observe the performance for the intervals between the transmission of 1 ms, 10 ms, and 100 ms. The OWD times obtained with gigabit Ethernet technology were 12 ms in the D2D operating mode, configured with an interval of 1 ms between messages. We observed an increase in OWD in all tests performed with Wi-Fi, with times between 15 ms for an interval of 1ms, reaching values close to 20 ms for the interval of 100 ms between messages. We also conducted experiments to evaluate the D2DxE mode, where tests with gigabit Ethernet fronthaul showed better performance, reaching 20 ms with the federation composed of two Helix brokers.

The second set of experiments carried out in the city of Aveiro had the presence of competing applications and traffic in the edge and core components. This condition made it possible to observe the behavior of Helix brokers and the impact on operating modes.

The experiments showed a slight increase in OWD in the D2D and D2DxE modes, respectively, with a mean latency of 15 ms and 23 ms. The results suggest that the presence of concurrent processes on IoT devices and APUs negatively impacts latency. In the D2DxCo mode, we observed a significant increase in latency when communication mediated by Helix brokers and CCIM used a 1 ms interval between messages. In other cases, latency remained at acceptable values. The D2DxC mode evidenced the platform’s ability to interconnect NGSI-based IoT devices in different countries with typical Internet latency.

Finally, we validated the ability of the Helix Multi-layered platform to manage complex context information generated by spectral probes at the edge, a function performed in the cloud by the Aveiro Tech City Living Lab platform.

The Helix Multi-layered platform enables a holistic view of context information distributed across cloud, fog, and edge layers. It has expanded the ability to process, merge, and disseminate context information across the available infrastructure, extending the use of standardized data models, complex entity relationships, conditional subscriptions, geolocation, and IoT agents to the lower levels of the network. The operating modes leverage device-to-device communication mediated by Helix brokers located in central datacenters and end-to-end computing structures of the continuum, enabling edge-to-edge, edge-to-cloud, fog-to-cloud, and cloud-to-cloud communication. The Helix platform emerges as a backend platform capable of integrating the industry ecosystem with the infrastructure of smart cities through open standards.

As future work, we plan to implement and test the device-in-transit (DT) operating mode with IoT devices installed on cars and buses with support to geolocation facing ITS applications. In the DT operating mode, the platform provides for the operation of devices that are in transit, that is, that eventually migrate from a specific coverage area or domain. The DT operating mode uses the transient entities (TE) feature that temporarily allows entities or devices to associate with the nearest context broker. The disfellowshipping process carries out automatically as soon as it reaches the expiration time. It uses the one-shot subscription mechanism to ensure notification to both devices and entities as long as they remain associated with the respective broker.

Finally, we aim to experiment with persistent connections across all brokers in the continuum, balancing performance and processing requirements. To reach this, it will be necessary to develop a solution to allow the switch between persistent and non-persistent notification connections during runtime. We also intend to analyze and manage CPU usage on IoT devices and edge computational elements for critical applications as the use of concurrent processes on these elements suggests performance degradation in real environments.

## Figures and Tables

**Table 1:** Synthesis of results—OWD for each operating mode and update requests interval.

Operating	Requests	OWD	OWD Standard	OWD
Mode	Interval	Mean (ms)	Deviation (ms)	Median (ms)
	1 ms	15.2	2.3	14.5
D2D	10 ms	16.1	2.0	15.8
	100 ms	18.2	2.1	17.8
	1 ms	21.7	4.6	20.5
D2DxE	10 ms	21.6	3.1	20.8
	100 ms	23.7	3.3	23.0
	1 ms	8315.9	5838.7	7066.6
D2DxCo	10 ms	63.8	43.1	49.8
	100 ms	30.3	4.6	29.7
	1 ms	454.5	12.7	451.1
D2DxC	10 ms	448.0	5.3	447.4
	100 ms	453.4	7.7	452.8
